# Drought-Induced Oxidative Stress in Pearl Millet (*Cenchrus americanus* L.) at Seedling Stage: Survival Mechanisms through Alteration of Morphophysiological and Antioxidants Activity

**DOI:** 10.3390/life12081171

**Published:** 2022-07-31

**Authors:** Shuvasish Choudhury, Debojyoti Moulick, Dibakar Ghosh, Mohamed Soliman, Adel Alkhedaide, Ahmed Gaber, Akbar Hossain

**Affiliations:** 1 Plant Stress Biology and Metabolomics Laboratory, Department of Life Science and Bioinformatics, Assam University, Silchar 788011, India; drubha31@gmail.com; 2 ICAR-Indian Institute of Water Management, Bhubaneswar 751023, India; dibakar.ghosh@icar.gov.in; 3 Clinical Laboratory Sciences Department, Turabah University College, Taif University, Taif 21995, Saudi Arabia; mmsoliman@tu.edu.sa (M.S.); a.khedaide@tu.edu.sa (A.A.); 4 Department of Biology, College of Science, Taif University, Taif 21944, Saudi Arabia; a.gaber@tu.edu.sa; 5 Department of Agronomy, Bangladesh Wheat and Maize Research Institute, Dinajpur 5200, Bangladesh

**Keywords:** antioxidants, drought, oxidative stress, pearl millet, redox implications, ROS

## Abstract

We report the impact of drought stress on pearl millet during the early seedling stage and its survival mechanism. Drought stress imposed for a period of 7, 14 and 21 days showed considerable changes in morphophysiological attributes, which were evident by a decline in seedling elongation, fresh and dry biomass, and relative water content (RWC) and degradation of chlorophyll pigment. Besides this, visible chlorosis lesions were observed in leaves as compared to the control. As compared to the respective controls, a nearly 60% decline in chlorophyll content was recorded after 14 and 21 days of drought stress. In both root and shoot, drought stress raised the reactive oxygen species (ROS) levels. Both H_2_O_2_ and O_2_^●^^−^ levels were significantly elevated along with a significant increase in lipid peroxidation in both roots and shoots, which clearly indicated ROS-induced oxidative stress. Concomitant with the increase in ROS levels and malondialdehyde (MDA) content in roots, membrane integrity was also lost, which clearly indicated ROS-induced peroxidation of membrane lipids. The activities of antioxidant enzymes and levels of non-enzymatic antioxidants were significant (*p* ≤ 0.001). After 7, 14 and 21 days of drought stress, activities of all the antioxidant enzymes viz., catalase (CAT), guaiacol peroxidase (GPX), superoxide dismutase (SOD) and glutathione reductase (GR) were inhibited, clearly indicating a loss of antioxidant defense machinery. Likewise, the levels of ascorbate (AsA) and reduced glutathione (GSH) levels declined significantly (*p* ≤ 0.01). Our results reveal that, being tolerant to arid climatic conditions, pearl millet is highly susceptible to drought stress at the early seedling stage.

## 1. Introduction

The utmost impact of climate change has resulted in the alteration of global precipitation patterns, either causing the emergence of recurrent droughts or excessive floods in many regions of the world. Today, among the other constraints present in the agro-environmental system (like heavy metals, salinity, etc.) drought is one of the most devastating abiotic stresses that strongly affect agriculture and threaten global food security [1,2,3,4,5,6,7]. The emergence of recurrent droughts has resulted in the desertification of agricultural lands and often rendering it unusable for a prolonged period. Further, the incidences of drought may likely increase progressively in the coming years in major food-producing regions of the world as a consequence of climate change, which will have a direct and stringent impact on agriculture and crop productivity [7,8]. The severity of drought on crop productivity depends mainly upon its duration and intensity [5,7,9]. Besides climate change patterns mainly influencing drought worldwide, other factors such as uncontrolled deforestation and related anthropogenic activities also contribute significantly to converting many productive areas into arid and drought-prone zones [7,10]. With the increase in the global population in the last few decades, food security is emerging as a major global crisis and droughts are contributing significantly to it as compared to any other abiotic factors [11,12]. The primary effect of drought stress in plants is the inhibition of seed germination due to the lack of moisture in the soil [13,14,15,16]. During drought, the osmotic balance is disturbed, reduction in turgor pressure causes impaired plant growth [14,17,18,19,20,21], reduction in root and shoot biomass, reduced leaf area and affects the overall growth and development [21,22,23,24]. Studies have demonstrated that drought stress results in altered mitosis, causing the cessation of cell expansion and elongation, which eventually affects crop yield [14,24].

Abiotic stresses are largely accompanied by an imbalance in cellular redox homeostasis. This arises mainly due to the overproduction of reactive oxygen species (ROS). The ROS are produced mainly due to the failure of antioxidant defense metabolism to counteract ROS overproduction under the environmentally stressed condition in a wide range of crops [25,26,27,28,29]. Drought stress imposes oxidative stress load on the cellular system by inducing such overproduction of ROS [30,31]. ROS entities such as hydrogen peroxide (H_2_O_2_), superoxide radical (O_2_^●^^−^), singlet oxygen (^1^O_2_) and hydroxyl radical (OH^●^) are highly reactive chemical entities, capable of reacting with cellular components and interfering with normal physiological and metabolic functions, and imbalance the cellular redox homeostasis [32,33,34]. Exposure to drought stress causes ROS-induced damage in plants; besides, ROS also act as signalling molecules to regulate diverse cellular responses [28,30,31]. Drought responses in plants involve complex traits, which can be achieved either by means of drought avoidance or tolerance [35].

Cereal crops such as pearl millet are widely grown in the arid agro-ecosystem of Africa and Asia. The crop is well known for its resistance against drought and high temperature in comparison to other cereals such as rice, barley, sorghum and wheat [36,37].

The investigation aims to study the impact of drought at the early seedling stage of pearl millet. Besides being well suited to arid conditions, the morphophysiological responses and drought-induced oxidative stress responses at early growth stages are not consistently known in this cereal crop, the basis of redox metabolism in pearl millet is quite fragmented. Thus, to ascertain this, the morpho-physiology, oxidative stress responses and antioxidant metabolism were evaluated after 7, 14 and 21 days of drought stress imposition in 2 days old pearl millet seedlings.

## 2. Materials and Methods

### 2.1. Plant Material and Drought Stress Imposition

The seeds of Pearl millet (*Cenchrus americanus* (L.) Morrone] were procured from the local market of Rajasthan, India, and brought to the laboratory in sterile plastic bags. The seeds were surface-sterilized with 0.1% (*w/v*) mercuric chloride (HgCl_2_) solution, followed by repeated rinsing in sterile deionized water. Sterile seeds were transferred to sterile plastic trays and germinated over moistened paper towels for 24–48 h at 30 ± 2 °C. The germinated seeds were transferred to plastic pots containing a soil mixture composed of sand (50%), sieved clay soil (40%) and vermiculite (10%) and grown for 3 days over growth racks with a 16 h photoperiod at 32 ± 2 °C. After 2 days of growth, drought was imposed by withdrawing water supply for a period of 21 days, while controls sets were moderately watered at every 2 days interval. After 7, 14 and 21 days of drought imposition, the root and shoot were harvested for analysis.

### 2.2. Growth Responses, Chlorophyll Content and Relative Water Content (RWC)

To study the morphophysiological responses, plant growth responses were recorded in terms of root and shoot elongation, fresh biomass and dry biomass. The RWC was calculated as RWC (%) = [(FW − DW)/(TW − DW)] × 100. The chlorophyll content was measured as per the method suggested by Arnon [38]. The total chlorophyll (Chl_t_) content was calculated as Chl_a_ (mgL^−1^) = 12.7 A_663_ − 2.69 A_645_; Chl_b_ (mgL^−1^) = 22.9 A_663_ − 4.68 A_645_. Total chlorophyll was determined as Chl_t_ = Chl_a_ + Chl_b_.

### 2.3. ROS Production, Lipid Peroxidation and Loss of Plasma Membrane Integrity

The ROS production was measured by determining the levels of hydrogen peroxide (H_2_O_2_) as per the method of Sagisaka [39] and O_2_^●−^ production as per the method suggested by Elstner and Heupel [40]. The lipid peroxidation was measured by measuring the malondialdehyde (MDA) content in pearl millet root and shoot as per the method of Heath and Packer [41]. The loss of plasma membrane integrity was measured spectrophotometrically by assay of Evans Blue (EB) uptake, as suggested by Yamamoto et al. [42].

### 2.4. Activities of Antioxidant Enzymes

The activities of the antioxidant enzymes in pearl millet root and shoot were determined by measuring the activities of enzymes such as catalase (CAT) [EC 1.11.1.6], guaiacol peroxidase (GPx) [EC 1.11.1.7], superoxide dismutase (SOD) [EC 1.15.1.1] and glutathione reductase (GR) [EC 1.8.1.7]. The CAT and GPX activities were determined as per the method of Chance and Maehly [43]. The SOD activity was determined as suggested by Beauchamp and Fridovich [44], while the GR activity was measured as per the method of Smith et al. [45].

### 2.5. Determination of Non-Enzymatic Antioxidants

To determine the non-enzymatic antioxidants, the levels of ascorbate (AsA) and total glutathione [GSH] were determined in pearl millet root and shoot [46,47]. For extraction of the metabolites, 0.2 g of fresh tissue samples were grounded to fine powder using liquid nitrogen and homogenised with 5% (*w/v*) slufosalicylic acid. The homogenate was centrifuged at 12,000 g for 10 min at 4 °C and supernatant was obtained. The reaction mixture for AsA was comprised of 2 mL each of 2% (w/v) Na–molybdate (Na_2_MoO_4_) and 0.15 N sulphuric acid (H_2_SO_4_), followed by the addition of 1 mL each of 1.5 mM disodium hydrogen phosphate (Na_2_HPO_4_) and supernatant extract. The mixture was vortexed and incubated at 60 °C for 40 min in a water bath, cooled and centrifuged at 3000 g for 10 min. The absorbance was recorded at 660 nm. The reaction mixture for determination of GSH content was comprised of 0.5 M K–phosphate buffer (pH 7.2) containing ethylenediamine tetraacetic acid (EDTA), 0.6 mM 5,5`-dithiobis-2-nitrobenzoic acid (DTNB), 2 mM nicotinamide adenine dinucleotide phosphate (NADPH) and 1U yeast GR (Type II). The absorbance of the reaction mixture was recorded at 412 nm.

The spectrophotometric measurements were made with a UV–Visible Spectrophotometer (Lambda 35 UV-Vis Spectrophotometer, Perkin Elmer, Waltham, MA, USA).

### 2.6. Statistical Analysis

The data presented are the mean of three replicates ± SE. All the datasets were statistically analyzed using the Tukey–Kramer multiple comparison test to evaluate the significant difference among the treatments at *p* ≤ 0.05/0.01, either using Microsoft Excel 2007 (Microsoft Inc. Redmond, Washington, USA) or INSTAT (Ver 3.0, Chicago, IL, USA).

## 3. Results

### 3.1. Morphophysiological Attributes

**Figure 1:**
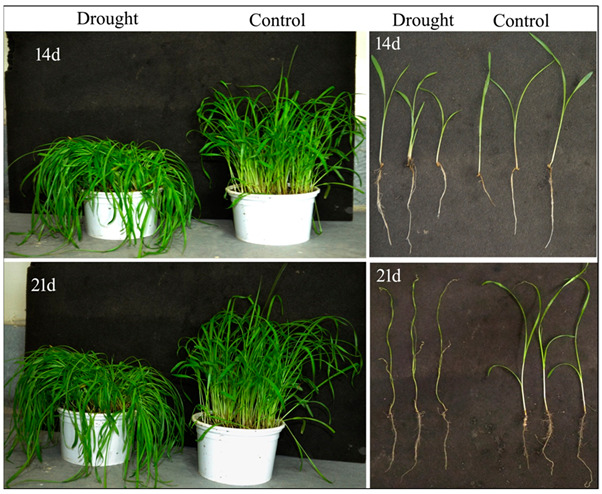
Impact of drought on pearl millet growth after 14 and 21 days of stress. Falling of leaves as a consequence of drought stress with respect to the controls was observed. Note: 14d and 21d indicate 14 and 21 days.

**Figure 2:**
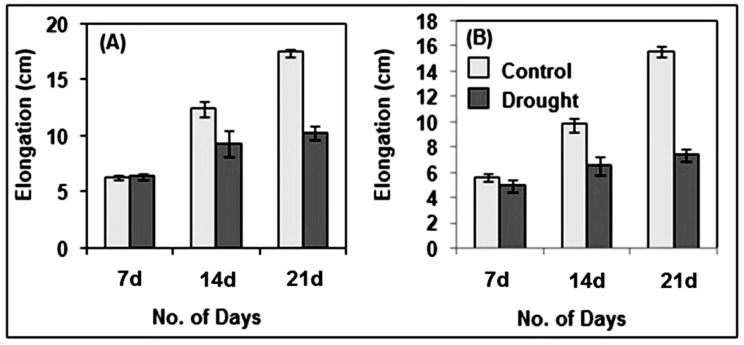
Effect of drought on the root (**A**) and shoot (**B**) elongation in pearl millet seedlings. The data presented are the mean of three replicates (*n* = 3), ±Standard Error (SE). Note: 7d, 14d and 21d indicate 7, 14 and 21 days.

The impact of drought stress on pearl millet was clearly observed in its morphological attributes such as growth, biomass and relative water content (RWC), along with changes in the total chlorophyll content. These attributes, when compared to the respective controls, showed a relatively significant impact of drought. Though drought stress did not alter the root or shoot growth (elongation) of pearl millet after 7 days of drought stress, a significant reduction in growth was observed after 14 and 21 days of stress imposition as compared to the controls (Figure 1 and Figure 2A,B).

**Figure 3:**
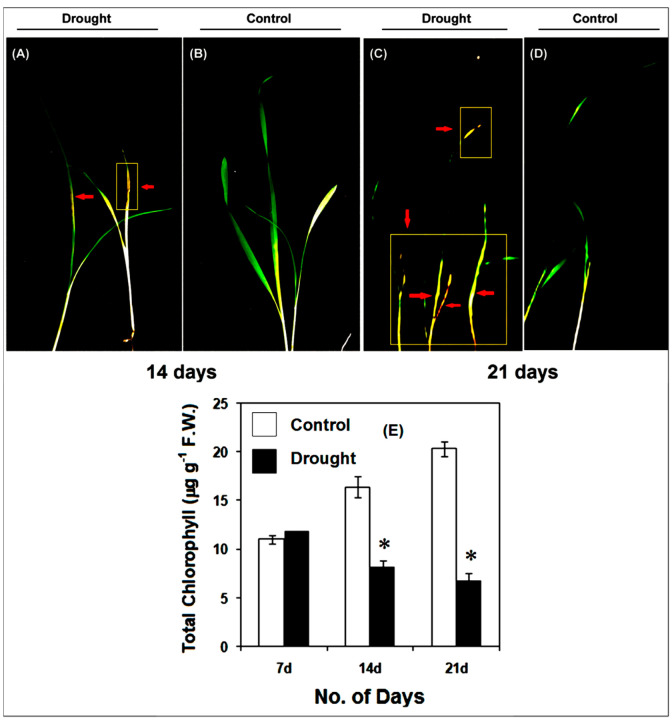
Drought induces changes in pearl millet seedlings after 14 (**A**,**B**) and 21 d (**C**,**D**) treatments, showing leaf curling and chlorotic lesions. (**E**) Shown is a decline in total chlorophyll content in pearl millet after 7, 14 and 21 days of drought stress. The data presented are the mean of three replicates (*n* = 3), ±Standard Error (SE). Asterisks (*) represent the significant difference at *p* ≤ 0.01 with respect to the controls. Note: 7d, 14d and 21d indicate 7, 14 and 21 days.

Along with a decline in root and shoot elongation, several noticeable drought-induced symptoms such as curling of leaves and strong necrotic lesions were also observed (Figure 3A–D). The total chlorophyll content also showed a significant decline after 14 and 21 days of drought stress as compared to the controls, which indicated a possible decline in photosynthetic efficiency as a consequence of drought stress (Figure 3E).

**Figure 4:**
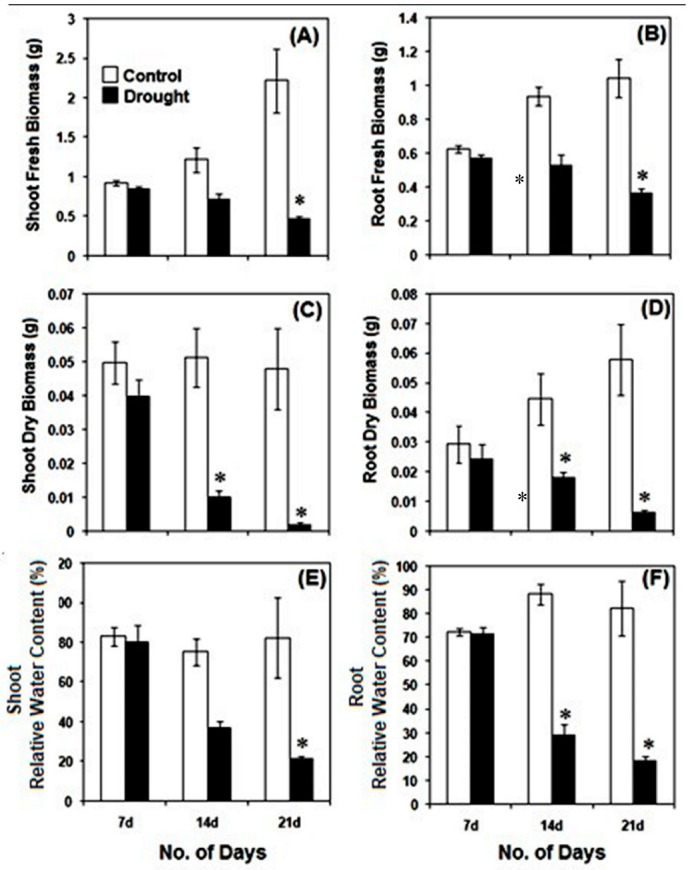
Drought-induced changes in fresh biomass (**A**,**B**), dry biomass (**C**,**D**) and relative water content (**E**,**F**) in pearl millet seedlings after 7, 14 and 21 days of treatment. The data presented are the mean of three replicates (*n* = 3), ±Standard Error (SE). Asterisks (*) represent the significant difference at *p* ≤ 0.01 with respect to the controls. Note: 7d, 14d and 21d indicate 7, 14 and 21 days.

After 7 days of drought, pearl millet seedlings apparently have similar chlorophyll content as that of the controls seedlings. However, with the drought conditions continued, a gradual and significant (*p* ≤ 0.01) decline in the chlorophyll content was observed. After 14 and 21 days of drought, leaves of pearl millet showed almost 2- and 3-fold less chlorophyll content as compared to the respective controls. The fresh and dry biomass of pearl millet seedlings declined significantly (*p* ≤ 0.01) after 14 and 21 days of drought stress as compared to the controls (Figure 4A–D). The impact of drought on root and shoot biomass was considerably noticeable after 14 days of drought stress, with a strong decline in biomass after 21 days. Under drought, the RWC was strongly affected in both root and shoot of pearl millet after 14 and 21 days of stress imposition, with practically no changes after 7 days of drought as compared to the controls (Figure 4E,F).

### 3.2. Drought-Induced Biomarkers

**Figure 5:**
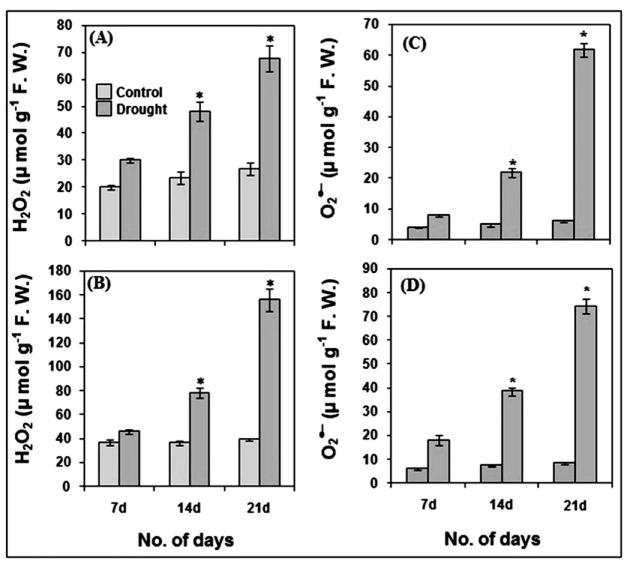
Production of reactive oxygen species (ROS) viz., hydrogen peroxide (H_2_O_2_) (**A**,**B**) and superoxide radical (O_2_^●−^) (**C**,**D**) in pearl millet after 7, 14 and 21 days of drought stress. The data presented are the mean of three replicates ±Standard Error (SE). Asterisks (*) represent the significant difference at *p* ≤ 0.01 as compared to the respective controls. Note: 7d, 14d and 21d indicate 7, 14 and 21 days.

**Figure 6:**
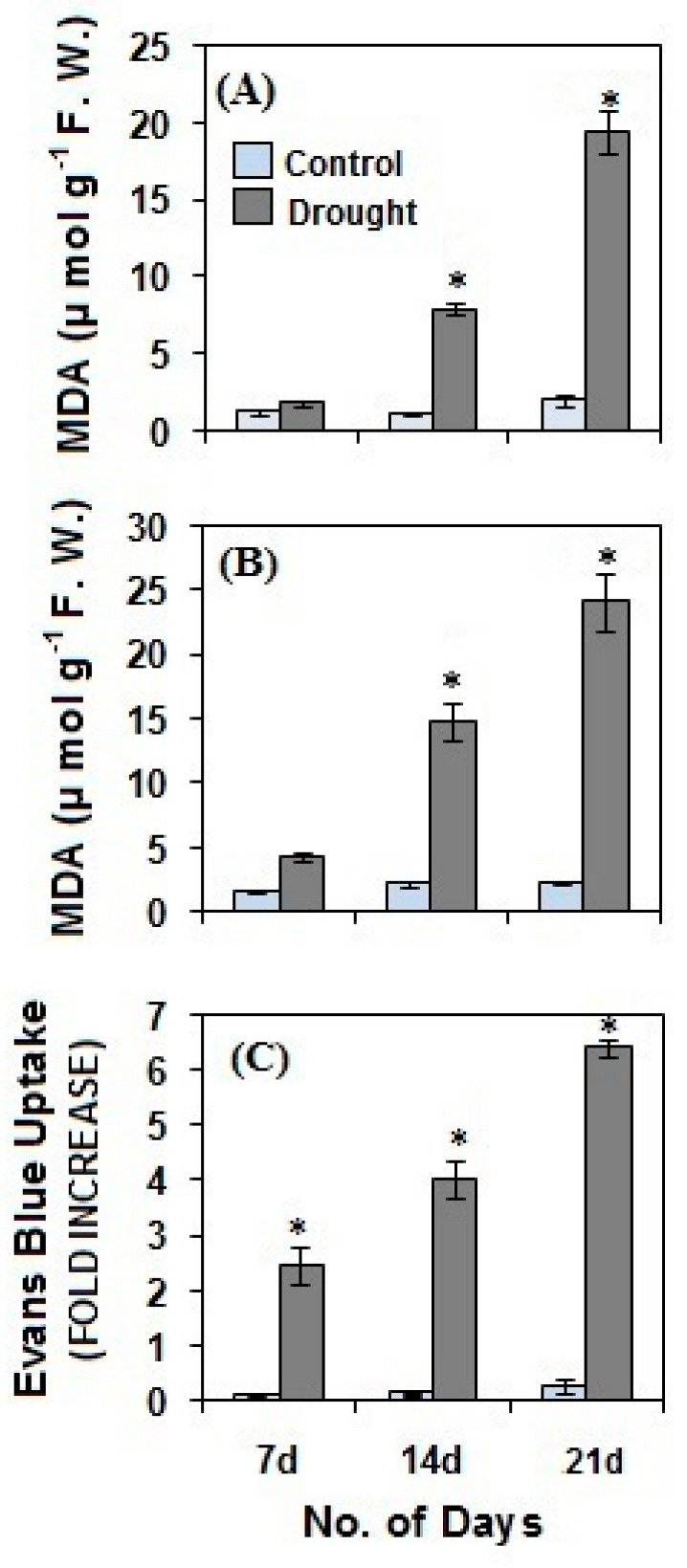
The onset of lipid peroxidation in pearl millet shoot (**A**) and root (**B**) and loss of root plasma membrane integrity (**C**) after drought stress. The data presented are the mean of three replicates ±Standard Error (SE). Asterisks (*) represent the significance level at *p* ≤ 0.01 as compared to the controls. Note: 7d, 14d and 21d indicate 7, 14 and 21 days.

Drought (stress)-induced elevated production of ROS such as changes in H_2_O_2_ and O_2_^●−^ (Figure 5A–C), and associated oxidative stress markers such as lipid peroxidation (Figure 6A,B) and loss of plasma membrane integrity in roots (Figure 6C), can be observed in pearl millet seedlings after 7, 14 and 21 days of drought stress imposition. The H_2_O_2_ content increased significantly (*p* ≤ 0.01) in both root and shoot after 14 and 21 days of stress as compared to the controls (Figure 5A,B). After 21 days of stress, H_2_O_2_ content was also significantly high (*p* ≤ 0.01) in both root and shoot as compared to 7 and 14 days of drought stress. Likewise, there was a significant increase (*p* ≤ 0.01) in O_2_^●−^ levels in shoot and root, respectively, of pearl millet seedlings as compared to the controls after 14 and 21 days of drought stress (Figure 5C,D). When the O_2_^●−^ levels during stress periods of 7 and 14 days were compared with those after 21 days, significantly (*p* ≤ 0.01) higher levels of the O_2_^●−^ content were observed in both shoot and root. The O_2_^●−^ content after 14 days was also significantly high (*p* ≤ 0.01) as compared to those after 7 days.

As a marker of oxidative stress, the malondialdehyde (MDA) content in pearl millet shoot and root (Figure 6A,B) was determined. A progressive and significant (*p* ≤ 0.01) increase in MDA content was observed in both shoot and root after 14 and 21 days of drought stress as compared to the controls. The results were concomitant with the rise in ROS levels, which induced a significantly high level of lipid peroxidation during drought stress. Further, we also assessed the plasma membrane integrity of pearl millet roots (Figure 6C) during drought stress by observing the fold increase in the uptake of Evan’s blue dye. Drought stress for the periods of 7, 14 and 21 days showed a significant (*p* ≤ 0.01) increase in the uptake of the dye as compared to the controls, which indicated that drought has resulted in a loss of plasma membrane due to ROS-induced peroxidation of plasma membrane lipids.

### 3.3. Antioxidant Metabolism

**Figure 7:**
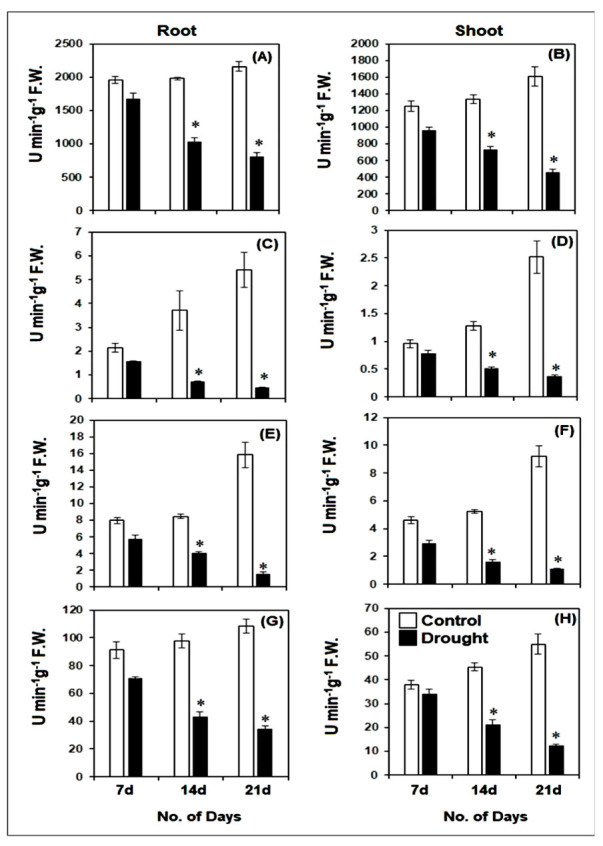
Changes in activities of catalase [CAT] (**A**,**B**), Guaiacol peroxidase [GPx] (**C**,**D**), glutathione reductase [GR] (**E**,**F**) and superoxide dismutase [SOD] (**G**,**H**) in root and shoot of pearl millet seedlings after 7, 14 and 21 days of drought stress. The data presented are the mean of three replicates ±Standard Error (SE). Asterisks (*) represent the significant difference at *p* ≤ 0.01 as to the respective controls. Note: 7d, 14d and 21d indicate 7, 14 and 21 days.

**Figure 8:**
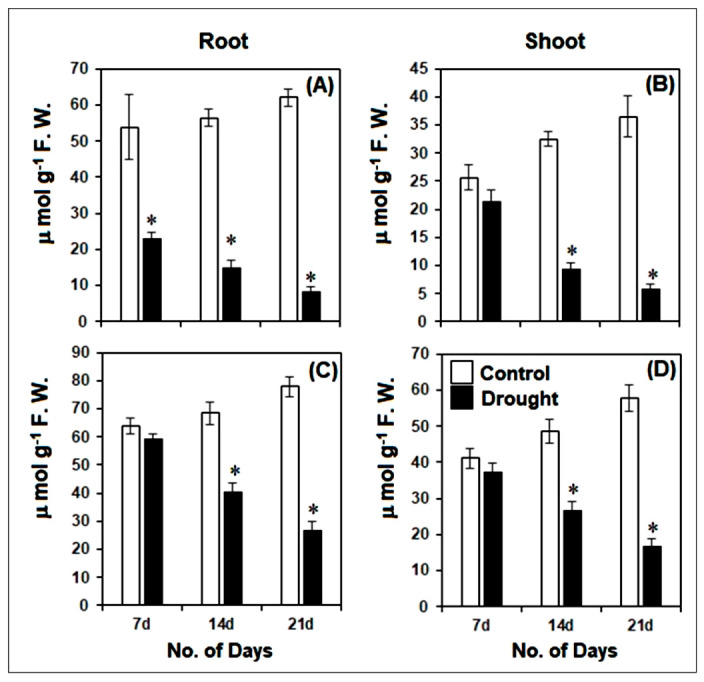
Changes in ascorbate [AsA] (**A**,**B**) and total glutathione [GSH] (**C**,**D**) levels in root and shoot of pearl millet seedlings after 7, 14 and 21 days of drought stress. The data presented are the mean of three replicates ±Standard Error (SE). Asterisks (*) represent the significant difference at *p* ≤ 0.01 as to the respective controls. Note: 7d, 14d and 21d indicate 7, 14 and 21 days.

Apart from the morpho-physiological perspective, there has been a significant influence of drought stress on antioxidant metabolism (Figure 7 and Figure 8). 

Drought stress for a period of 7, 14 and 21 days significantly (*p* ≤ 0.01) affected the activities of enzymatic antioxidants such as CAT, GPx, GR and SOD in both root and shoot (Figure 7A–H). In comparison to their respective controls, the activities of these enzymes were gradually inhibited with the increase in the duration of drought. As compared to the 7 days old drought-stressed seedlings, the CAT activity was inhibited by almost 38% and 52%, respectively, after 14 and 21 days of stress in roots, while in shoot the CAT activity was inhibited by almost 23% and 52% after 14 and 21 days of stress, respectively, as compared to those after 7 days of drought stress. The GPx activity in roots was strongly inhibited by almost 54% and 70% after 14 and 21 days as compared to 7 days of drought stress. A similar trend was also observed in shoots, where GPx activity was inhibited by nearly 33% and 55% after 14 and 21 days, respectively, as compared to 7 days of drought stress. Similarly, the activity of GR in roots was inhibited by 29% and 72% of roots after 14 and 21 days of drought stress, respectively, while GR activity in the shoot was inhibited by 45 and 62% after 14 and 21 days of drought stress, respectively, as compared to the GR activity in root and shoot after 7 days of drought. The SOD activity in pearl millet root was inhibited by almost 39 and 51%, respectively, as compared to those after 7 days of drought stress. In shoots, SOD activity was inhibited by nearly 37 and 64%, respectively, after 14 and 21 days as compared to 7 days of drought stress. The decline in the activity of these enzymes clearly indicated the strong deleterious impact of high levels of ROS in the root and shoot of pearl millet seedlings under drought stress.

The levels of non-enzymatic antioxidants such as AsA and GSH declined significantly (*p* ≤ 0.01) in pearl millet root and shoot as compared to the controls (Figure 8A–D). After 21 days, the AsA and GSH levels declined by almost 86% and 65% in respective roots, while nearly 84 and 70% declines in AsA and GSH levels were observed in shoot, respectively, as compared to the controls. Amongst the drought-stressed seedlings, the AsA levels declined by almost 34 and 63% in respective roots after 14 and 21 days as compared to 7 days of drought stress. In the shoot, the AsA levels declined by 56% and 72%, respectively, after 14 and 21 days as compared to 7 days of drought stress. The trend of decline in GSH levels was similar to AsA under drought for 14 and 21 days as compared to 7 days of drought-stressed pearl millet seedlings. In roots, the GSH levels were reduced by nearly 32 and 54% after 14 and 21 days, respectively, as compared to 7 days of drought. In the case of shoot, the GSH levels after 14 and 21 days of drought stress were reduced by 28% and 54%, respectively, as compared to 7 days of drought-stressed pearl millet seedlings.

## 4. Discussion

As an impact of climate change, the majority of agroclimatic conditions are altered and affected by diverse abiotic stresses. Drought is considered to be the major contributing factor to the decline in crop productivity and yield, thus threatening global food security. The scarcity of water from an agricultural perspective is a global concern and intensified with every passing day. Among grain crops, pearl millet is ranked at the sixth position and is widely cultivated in India, covering an agricultural area of close to 7.0 million ha, with an average annual production of almost 9 million tons [48]. With the ever-increasing impact of drought, especially on cereal crops, in-depth insight into physiological and biochemical responses of pearl millet under drought stress is attempted in the present investigation.

Subjected to drought stress after 7 days of growth under normal conditions till 21 days, pearl millet seedlings showed significant alterations in their morpho-physiology, redox metabolism, oxidative stress and antioxidant metabolism in root and shoot. Compared to the controls, drought-exposed seedlings have significantly less root and shoot elongation, reduction in fresh or dry biomass and highly altered RWC. These parameters were greatly impacted after 14 and 21 days of drought. Under drought conditions, the disposition of these traits exhibited considerable susceptibility of pearl millet to drought stress, and, as such, these can be considered useful datasets for comprehensively improving drought stress adaptation [49]. Further, our findings also showed the pattern of root growth (vertical and lateral), which indicated an effort to penetrate deeper into the soil for the search for available moisture [50]. In the absence of an external water supply under laboratory conditions, the soil drying rate was ominously high, thus affecting the root and progressively shoot RWC. Drought stress also affected the photosynthesis of pearl millet seedlings, as indicated by a significant decline in total chlorophyll content. Photosynthesis is considered an essential metabolic process, which is known to be affected by a variety of stresses, including drought. With a significant reduction in chlorophyll content with practically no phyto-availability of water, the overall photosynthetic process is substantially hampered, leading to low biomass and growth [51,52,53].

The impact of drought on pearl millet can also be observed by significant changes in redox metabolism and the onset of oxidative stress. Significantly high accumulation of ROS such as H_2_O_2_ and O_2_^●−^ were observed in both root and shoot of pearl millet seedlings under drought stress. High ROS production is accompanied by an increase in MDA content, which clearly indicated oxidative stress. Further, in roots, the increase in the uptake of Evans blue dye indicated the loss of plasma membrane integrity, which is the consequence of membrane lipid peroxidation. ROS are inevitable entities of aerobic life. As a consequence of drought, the rate of photosynthesis decreases and ROS production can increase by several folds, leading to an oxidative burst [30,54,55]. Thus, drought stress disrupts the cellular redox homeostasis by increasing the levels of ROS and enhancing the process of lipid peroxidation, loss of plasma membrane integrity, altered stomatal function, growth retardation, early senescence and ultimately results in poor crop yield [56].

The response of both enzymatic and non–enzymatic antioxidants in pearl millet seedlings under drought stress clearly reflected the loss of cellular redox balance. The activities of all the antioxidant enzymes studied, such as CAT, GPX, GR and SOD, were found to be significantly affected by drought. Likewise, the levels of non-enzymatic antioxidants such as AsA and GSH were significantly lowered in both the root and shoot of pearl millet seedlings. Although antioxidants function to scavenge the ROS and maintain the cellular redox homeostasis, severe drought stress imposed for a period of 21 days at the seedling stage disrupted the antioxidant metabolism in pearl millet. In the absence of CAT and GPx activities in pearl millet due to drought stress, the levels of H_2_O_2_ increased progressively. Concomitant with the rise of H_2_O_2_ levels, the O_2_^●−^ levels also increased significantly due to the degradation of SOD activity. All these enzymes together serve as frontline antioxidants to scavenge H_2_O_2_ and O_2_^●−^ in order to maintain proper redox balance [27,57]. With an enormous load of ROS, the levels of non-enzymatic antioxidants such as AsA and GSH declined significantly in pearl millet seedlings under drought stress. The sharp decline in the levels of AsA and GSH clearly indicated a comprehensive loss of an antioxidant defense mechanism as the duration of the drought was increased in pearl millet seedlings.

The findings of the present study reflect physiological variations and oxidative stress responses in pearl millet subjected to drought stress at an early seedlings stage. With a minimum requirement of water, pearl millet is largely cultivated in arid agro-ecosystems. Although pearl millet can withstand prolonged water-deficit conditions, at an early seedling stage, drought can turn highly injurious and threatens its survival. An increase in ROS levels and failure of antioxidative metabolism leads to the onset of oxidative stress, causing considerable alterations in cellular homeostasis and disrupting normal cellular functions.

## 5. Conclusions

Although pearl millet is considered naturally tolerant to semi-arid and arid climatic conditions, complete withdrawal of irrigation leads to a serious deleterious impact on its morphological attributes, redox metabolism and antioxidant defense system, thus disrupting the cellular and functional homeostasis. In this study, drought was simulated for a period of 21 days at an early seedling stage of pearl millet, which resulted in high production of ROS and caused complete deterioration of antioxidant defense metabolism. Our study revealed that, at the early seedling stage, pearl millet is highly susceptible to drought stress.

## Data Availability

Not Applicable.
